# Retrospective Analysis of the Real-World Use of Topical Antimicrobials in the Paediatric Population with Impetigo in Italy: Focus on the Role of Ozenoxacin 1% Cream

**DOI:** 10.3390/children10030547

**Published:** 2023-03-13

**Authors:** Elisa Barbieri, Sara Cavagnis, Riccardo Boracchini, Antonio Scamarcia, Angela Testa, Maria Grazia Ciarniello, Biancangela Martinelli, Luigi Cantarutti, Carlo Giaquinto, Anna Cantarutti

**Affiliations:** 1 Division of Pediatric Infectious Diseases, Department for Woman and Child Health, University of Padua, 35128 Padua, Italy; 2 Società Servizi Telematici—Pedianet, 35100 Padua, Italy; 3 Unit of Biostatistics, Epidemiology and Public Health, Department of Statistics and Quantitative Methods, University of Milano-Bicocca, 20126 Milan, Italy; 4 National Centre for Healthcare Research and Pharmacoepidemiology, Department of Statistics and Quantitative Methods, University of Milano-Bicocca, 20126 Milan, Italy; 5 Angelini Pharma S.p.A., 00181 Rome, Italy

**Keywords:** topical antibiotics, children, impetigo, ozenoxacin, Pedianet database, Italy

## Abstract

Using electronic data from a large population-based network of Family Paediatricians (Pedianet), we aimed to describe the use of topical antimicrobials, including ozenoxacin 1% cream, in impetigo in children in Italy. We included 2929 children aged 6 months–14 years from 2016 to 2019 with at least one episode of impetigo treated with topical antimicrobials. Overall, 3051 cases of impetigo were included in the analysis. Treatment started in most cases on the same day as the impetigo diagnosis and lasted around eight days. In about 8% of the cases, a systemic antibiotic was prescribed after the topical antimicrobial, usually after 4–14 days. In this study, ozenoxacin was used in 8% of the cases. Treatment duration was significantly shorter for patients prescribed ozenoxacin compared to the whole study population (median of six vs. seven days, respectively). In contrast, the rate of treatment failure was similar. Very few adverse reactions were identified.

## 1. Introduction

The resistance rates of methicillin-resistant *Staphylococcus aureus* (MRSA) strains isolated from blood and cerebrospinal fluid in 2021 Italy was 29% [1], doubling the median resistance rate in Europe [2]. Even if no increasing trend has been recorded over the years, the diffusion of MRSA strains in the community setting (CA-MRSA) has become a public health concern [1,3].

Consequently, soft-skin and tissue infections caused by CA-MRSA, including impetigo, may be more difficult to treat [3,4,5,6] with relatively fewer antibiotic agents available to treat CA-MRSA infections in pediatric primary care, including trimethoprim/sulfamethoxazole, tetracyclines, doxycycline, minocycline and clindamycin (for osteomyelitis) [7]. Moreover, the available agents have substantial limitations related to the pharmaceutical form that might not be well suited to treat small children in the primary care setting and the need for off-label use because of age limitation in some cases (i.e., doxycycline). Finally, the development of new antibiotics has slowed over the years, further diminishing future possibilities to tackle CA-MRSA [6,7].

Ozenoxacin is a quinolone antibiotic used to treat impetigo in adults and children. It is particularly active against impetigo caused by *S. aureus* or *Streptococcus pyogenes* but has also been active against bacteria resistant to fluoroquinolone [8]. 

Since 2018 ozenoxacin 1% cream has been approved in 19 European countries in patients aged six months or older [9,10], while in the USA and Canada, ozenoxacin 1% cream is indicated for topical treatment of non-bullous and bullous impetigo in patients aged two months and older [11]. A recent study pooling data from several phase 1 and 3 studies showed that ozenoxacin was significantly more effective than a placebo vehicle in children with non-bullous impetigo (NBI) [12]. Still, the limitation of clinical trials in the generalizability of the results is well recognized. Indeed, the studied population is very different from the population treated in the current clinical practice; in our recent study on the burden of NBI in the Italian primary care setting [13], oral antibiotics were preferred over topical antibiotics (54.8% vs. 29.5%) even if not recommended in the most recent guidelines [14]. Regarding topical antibiotics, mupirocin, fusidic acid, and gentamycin were the most prescribed treatments for impetigo. 

So far, there is a paucity of up-to-date data on the real use of topical antimicrobials in the pediatric population in Italy [15,16]. Also, our previous study was limited to 2004–2018, and we could not assess the impact that ozenoxacin might have had on the children’s care, especially the youngest.

Using electronic data from a large population-based network of Family Paediatricians (Pedianet) we aimed to describe the use of topical antimicrobials, including ozenoxacin 1% cream, in impetigo, both bullous and non-bullous, in children in Italy.

## 2. Materials and Methods

### 2.1. Setting

The data used for the present study were retrieved from the electronic data from a large population-based network of Family Paediatricians (Pedianet) of Italy that accounts for about 3% of the Italian pediatric population [17]. Italy is one of few countries where a specific primary care system is devoted to children up to 14 years. Within the Italian National Health Service (NHS) framework, every child is registered at birth and receives free medical care from one of the approximately 5000 FPs who work for the NHS.

### 2.2. Data Source

Set up in 1999, Pedianet currently collects the clinical, demographic, and prescription data for children who have provided informed consent and are under the care of any family pediatricians (FPs) that currently provide data to the database in Italy. Data are generated during routine patient care with the software JuniorBit ^®^ and are stored in different files, which can be linked through a unique (anonymous) numerical identifier. The identification file contains information on the child’s demographic data and eligibility status (registration status, date of registration, and date of death). The prescription file contains information on all drugs (date of prescription, ATC code, product, quantity, dosing regimen, legend duration, indication, reimbursement status) and vaccinations that the FPs prescribe. Reasons for contact and diagnoses (free text or coded by the ICD9-CM system) are collected in the medical files, including all anthropometric measurements (weight and height). In addition, the database contains information on referrals to specialists, procedures, hospitalizations, medical exams, health status (according to the Guidelines of Health Supervision of the American Academy of Paediatrics), and centile diagrams. All patients provided written informed consent for their data to be collected and used for research purposes. The percentage of consent refusal is <5%, and there are no differences between families providing and not providing consent.

### 2.3. Cohort Selection

The target population consisted of children with at least one impetigo case treated with a topical antimicrobial. All children were aged <14 years, were enrolled from 1 January 2016 to 30 December 2021 with one of the FPs parts of the Pedianet network and had at least 30 days of follow-up from the impetigo diagnosis. 

Impetigo cases were identified with ICD9-CM codes (684 and 694.3) or free text (in Italian, “impetigine” and related abbreviations) in the diagnosis field. Cases were evaluated manually to exclude any false-positive cases by a clinical data manager. To avoid duplicates, medical records with the same diagnosis <30 days apart were considered as a follow-up of the initial case. Of these, cases that received at least one topical antimicrobial prescription were identified, and the first drug prescription was defined as the index date. 

All topical antimicrobial prescriptions associated with the same impetigo treatment episode being prescribed less than 14 days after a systemic antibiotic drug identified by the ATC field J01* (i.e., the difference between the J01 prescription date and the topical antimicrobial prescription date is <14) were excluded from the analysis. 

### 2.4. Exposure

The Pedianet database holds information on all prescribed prescriptions. Topical antimicrobial exposure was defined as a prescription for medicines with the Anatomical Therapeutic Code D01AC*, D06AA*, D06AX*, and D07*. 

### 2.5. Outcomes

The primary outcome of the study was treatment failure, defined as a second antimicrobial prescription with a different ATC (both topical and/or systemic) of the index prescription in a time frame of 14 days after the prescription index and defined as an early (days 1–3) or a late (days 4–14) failure.

The secondary outcomes of interest were: (i) the adverse effect within 30 days from the index date (i.e., itch, skin rash, dermatitis, eczema, edema, urticaria, angioedema, skin vesicular, pain at the application site, generic allergic reaction, hypertrichosis, hypopigmentation, skin atrophy), and (ii) the treatment duration defined based on the prescribed daily dose and expressed in days of treatment.

Adverse events were identified with ICD-9-CM codes (Table S1 in the Supplementary Materials) and by searching the free text of the diagnosis.

Treatment duration was calculated as the difference between the last prescription’s end and the first one’s start. Only drugs with prescribed daily doses reported were considered in this latter analysis. We considered a median prescribed daily dose of seven days for systemic antibiotics. Moroever, treatment duration was stratified by treatment with only topical antimicrobials and treatment with topical antimicrobials and systemic antibiotics. In addition, treatment duration was stratified by type of treatment (i.e., therapy with only topic drugs or topical and systemic).

Dermatological visits between 15–30 days after the index prescription were captured.

### 2.6. Covariates

Baseline characteristics included sociodemographic (i.e., age at the start of the study, sex, and geographic area (North, Center, South with Islands)) and clinical characteristics (i.e., the presence of chronic conditions, grouped by respiratory (asthma, bronchopulmonary dysplasia, wheezing), cardiovascular, diabetes, immunosuppression or immunosuppressive therapy (including cancer therapies), Down syndrome, and dermatological (i.e., psoriasis, atopic dermatitis, chronic urticaria, alopecia areata, vitiligo) (Supplementary Materials Table S2. Moreover, time to administration was reported as the difference in days between the diagnosis index date and the prescription index date.

### 2.7. Statistical Analysis 

The analysis was descriptive. The distribution of children’s sociodemographic and clinical characteristics was described among children. The treatment failure, overall and stratified by early and late, as well as the treatment duration, were defined among impetigo cases stratified in the analyses by type of antimicrobials (topical, with particular focus on the ozanexocin vs. sistemic antibiotic medications).

A detailed analysis was reported on treatment failure specifying all antimicrobial classes to evaluate which topical antimicrobials had the greatest treatment failure and if there was any difference following the approval of ozenoxacin.

All analyses were performed using the Statistical Analysis System Software (version 9.4; SAS Institute, Cary, NC, USA).

## 3. Results

Overall, 2929 children who had been diagnosed with at least one impetigo diagnosis from 1 January 2016 to 30 December 2021 and had been prescribed a topical antimicrobial were included in the study. A total of 3051 cases were identified: most children (N = 2813) had only one impetigo, with around 4% having two or more cases. In 8.4% of the cases (256/3051 cases), ozenoxacin was prescribed. (Table 1) The most prescribed topical antimicrobials (74%) were about the class of other topical antibiotics (ATC: D06AX*) followed by antibiotics in association with a corticosteroid (23%, ATC: D07C*). The distribution remains similar after the approval of ozanexozacin since the latter is part of the other topical antimicrobial class (Supplementary Materials Table S3). In the period before ozenoxacin was available on the market, fusidic acid was prescribed in 37.8% of the cases, followed by muciprocin (18%), antibiotics in association with betamethasone (17.2%), and gentamicin (13.1%); in the period after, the most prescribed was muciprocin (26%), followed by fusidic acid (20.8%) and ozenoxacin (20.3%) (Supplementary Materials Table S3). 

The mean age of the cases on the day of the diagnosis was 70.8 months (SD 43.4 months): almost 40% of the children were two years or younger, similar to those prescribed ozenoxacin. There were slightly more males than females with a diagnosis of impetigo (56% vs. 44% in the whole study population), and the majority of children lived in the northeast region of Italy. This variation is likely irrelevant and should be attributed to the different enrolment patterns of FPs in Pedianet (Table 1). 

A total of 96% of the population did not have a chronic condition. Of the 108 children with comorbidity, only 10 had more than one condition. The most common conditions were being born pre-term (48.4%, 62/128), having a respiratory disease (16.4%, 21/128), and having neurological and cardiovascular diseases (both 9.4%, 12/128). No chronic dermatological diseases were identified. (Table 2).

### 3.1. Treatment Duration and Time to Treatment Initiation

Dosage regimen details were reported for 51.72% of cases (1578/3051). The mean duration of treatment for topical antimicrobials was 7.48 days (SD 1.95 days), with values ranging from 2–21 days. The dosage regimen was reported in 32.81% of ozenoxacin cases (84/256). The mean treatment duration was 7.02 days (SD 1.86 days), with values ranging from 5–18 days. Overall, treatment duration is shorter when ozanexocina was prescribed at the index date and when only topical antimicrobials were used (Supplementary Materials Table S3). Regarding time to treatment initiation, in the majority of the cases, the topical antimicrobial was prescribed the same day of the diagnosis: the mean time from diagnosis to prescription was 0.04 days (SD 0.64), with a range of 0–14 days. Similar values were reported for ozenoxacin prescriptions (mean time to treatment initiation = 0.04 days, range 0–7 days).

### 3.2. Treatment Failure and Adverse Events

A total of 2911 (95.4%) cases were treated in monotherapy on the index date and 140 (4.6%) in polytherapy, with at least three different topical antimicrobials at baseline (3, 0.1%). Overall, a second prescription was more commonly made for a systemic antibiotic than a different topical antimicrobial. In 2% of the cases, a systemic antibiotic was prescribed within three days of the first prescription, and in 5% of the cases between 4–14 days after. In five cases, two separate systemic antibiotic prescriptions were prescribed in the early and late timeframe (Table 3). 

Late treatment failure among topical antimicrobials was more common, with 0.6% of the cases being prescribed different topical antimicrobials after 4–14 days from the first prescription (Table 3).

Regarding ozenoxacin, the rate of prescriptions of a systemic antibiotic was similar to all prescriptions; late failure was more frequent. There wasn’t a prescription for a different topical antimicrobial (Table 3).

Most of the treatment failures were identified after a prescription of fusidic acid (D06AX01), gentamicin (D06AX07), and thyrotricin (D06AX09). About 45% of treatment failures were replaced with amoxicillin and clavulanic acid, 11% with azithromycin, and 9.6% with amoxicillin (Table 4). Treatment switches were in the ozenoxacin group.

0.13% of the cases presented an adverse event (4/3051): Three cases presented vomiting, and one presented vomiting and diarrhea. Three adverse events were recorded after the assumption of fusidic acid, and one vomiting reaction was recorded after the assumption of a combination of gentamicin and triamcinolone + an antibiotic. No adverse events were recorded after an ozenoxacin prescription.

## 4. Discussion

In this study, we analyzed 3051 cases of impetigo treated with topical antimicrobials. Treatment started in most cases on the same day of the impetigo diagnosis and lasted around eight days. In about 7% of the cases, a systemic antibiotic was prescribed after the topical antimicrobial, usually after 4–14 days. Very few adverse reactions were identified. When treatment failure occurred, the most used were penicillins with or without a betalactamase inhibitor.

Overall, there is very limited evidence on real-world treatment of impetigo, particularly in the primary care setting. The results of a Dutch study published in 2019 are generally consistent with this study. The Authors reported that most cases were treated at the time of the diagnosis [18]. In around 15% of the cases, there was a second prescription for a different antibiotic; this is almost twice the rate of treatment failure reported in our study (8% of the cases). This difference might be attributed to the different population selections of the two studies since, in the Dutch paper, people of all ages were included. In both the Dutch study and this report, the clinician prescribed systemic antibiotics more frequently as subsequent treatment rather than topical antibiotics. This aligns with international and Italian guidelines on impetigo [13,19], which recommend systemic treatment in case of poor response to the first topical treatment.

There is even less evidence available on ozenoxacin, which consists mainly of clinical trials (including [20,21,22]). In this study, ozenoxacin was used in 8% of the cases, and its use seemed to increase with time, as the age at the start of the study was smaller for children who were later prescribed ozenoxacin. Treatment duration was slightly shorter for patients prescribed ozenoxacin compared to the whole study population (7.02 vs. 8.02 days), while the treatment failure rate was similar. It should be noted that the EMA’s approved duration of treatment is five days [8], significantly shorter than the duration of treatment reported in this study. A Spanish study published in 2022 yielded slightly different results. The authors reported that impetigo cases treated with ozenoxacin had a shorter duration of treatment, a lower rate of complications, and a lower average cost compared to cases treated with fusidic acid and mupirocin. However, in this study, the analysis was not limited to pediatric patients (15% of the cases were 18 or older), which makes the comparison with our study harder to interpret [23].

Adverse reactions were overall uncommon (≥1/1000 to <1/100) and most reported for fusidic acid. These results are consistent with reports of mainly mild gastrointestinal side effects for fusidic acid [24,25]. As highlighted in the results, our methods to identify adverse reactions could have led to an underestimation of ARs; however, it is very unlikely that any non-reported AR was of a serious nature.

The main strength of our study is the nature of the data source: Pedianet collects information from family pediatricians throughout Italy and has a representative coverage of Italian pediatric patients [16]. The study population is, therefore, representative of children with impetigo attending primary pediatric care. Also, as noted earlier, there is a lack of evidence on the real-world use of ozenoxacin and topical antimicrobials for impetigo [13], and this study begins to address this gap.

The study also has several limitations. We assessed prescriptions by the FPs, but it is possible that some cases were prescribed antimicrobials during a specialist dermatology visit; the rate of treatment failure could therefore be higher than what we estimated. It is also possible that some clinicians prescribed an antimicrobial but recommended parents wait 1–2 days to evaluate the natural clinical course of the disease before giving any medication. Moreover, as highlighted before, we assessed the treatment failure rate, but we did not consider whether there was a treatment switch (i.e., the two antibiotics could have been used concurrently), and it was not feasible to assess the reason for the prescription of a second antimicrobial. Furthermore, treatment failure will most likely be identified at the end of the antimicrobial course, while treatment switch in the first days after the prescription (early switch) will more likely be due to adverse events (including irritation and drug-drug interaction) or irrational prescribing practice. However, this cannot be ascertained for sure. Finally, treatment in the primary care setting, there is no requirement for microbiological testing to confirm a treatment failure. Most treatment failures are identified after an examination by the clinician. However, the practice changed after the COVID-19 pandemic, with telemedicine visits increasing 5-fold [14]. It is unclear how this might have impacted the pediatricians’ decision to prescribe a second antimicrobial.

## 5. Conclusions

Our study reported on the use of topical antimicrobials for a common skin infection in routine care; the results suggest similar rates of complications and treatment failures but a shorter duration of treatment, in impetigo cases treated with ozenoxacin, compared to other topical antimicrobials. In a time of increasing antimicrobial resistance, with the development of new antibiotics slowing down [26], research on the real-world use of antimicrobials is critical to planning and implementing stewardship programs. Further evidence on the possible causes of treatment failure is necessary, as well as on the impact of the pandemic on primary care treatment of skin infections.

## Figures and Tables

**Table 1:** Sociodemographic and clinical characteristics of the children included in the study (N = 2929 overall children, N = 254 children prescribed ozenoxacin). Pedianet, 2016–2021.

		Overall (Ntot Cases = 3051) N (%)	Ozenoxacin 1% Cream (Ntot Cases = 256) N (%)
Number of cases	1	2813 (96.04)	252 (99.21)
	2	110 (3.76)	2 (0.79)
	3	6 (0.20)	
Age at the start of the study	Mean (SD)	46.35 (41.18)	37.12 (37.37)
	0–6 months	658 (22.47)	81 (31.89)
	6 months–2 years	494 (16.87)	38 (14.96)
	2–5 years	833 (28.44)	71 (27.95)
	5–14 years	944 (32.23)	64 (25.20)
Sex	Male	1641 (56.03)	137 (53.94)
	Female	1288 (43.97)	117 (46.06)
Region	North-West	196 (6.69)	28 (11.02)
	North-East	1912 (65.28)	208 (81.89)
	Centre	322 (10.99)	17 (6.69)
	South	317 (10.82)	1 (0.39)
	Islands	182 (6.21)	
Chronic condition	None	2811 (95.97)	241 (94.88)
	1	108 (3.69)	11 (4.33)
	>1	10 (0.34)	2 (0.79)

**Table 2:** Chronic conditions grouped by type (N = 128). Pedianet, 2016–2021.

Comorbidity Group	N	%
Born pre-term	62	48.44
Respiratory	21	16.41
Neurological	12	9.38
Cardiovascular	12	9.38
Immunosuppressive therapies/immunodeficiencies	6	4.69
Diabetes	3	2.34
Down syndrome	1	0.78
Other	11	8.59
Total	128	100

**Table 3:** Treatment failure (N = 3051 overall cases, N = 256 cases prescribed ozenoxacin). Pedianet, 2016–2021.

Second Prescription Type	Timing	Overall		Ozenoxacin 1% Cream	
**Systemic antibiotic**		N	%	N	%
	No TF	2831	92.79	234	92.13
	Early	60	1.97	11	3.15
	Late	155	5.08	8	4.33
	Both early and late	5	0.16	1	0.39
**Topical antimicrobial**	No TF	3031	99.33	252	100
	Early	2	0.07	0	
	Late	18	0.6	0	

**Table 4:** Combination of topical and systemic therapies prescribed among treatment failure. Pedianet, 2016–2021.

		Second Prescription					
		Other Topical Antibiotics (%)	Betalactams/Penicillins (%)	Cephalosporins (%)	Macrolides (%)	Sulfonamides/Trimethoprim (%)	Total (%)
**First prescription (topical)**	**Ozanexocin 1% cream (%)**	-	5.52	8.51	15.09	-	7.02
	**Other topical antibiotics (%)**	-	69.94	72.34	66.04	100.00	77.59
	**Antibiotics with antimycotic properties (%)**	0.33	3.68	-	-	-	3.01
	**Antibiotics in combination with corticosteroids (%)**	-	19.02	17.02	18.87	-	17.73
	**Chlortetracycline (%)**	-	1.23	2.13	-	-	1.67
	**Total (%)**	0.33	54.52	15.72	17.73	1.00	

## Data Availability

The data analyzed in this study is subject to the following licenses/restrictions: The data used in this study cannot be made available in the article, the Supplementary Materials, or in a public repository due to Italian data protection laws. The anonymized datasets generated during and/or analyzed during the current study can be provided on reasonable request, from the corresponding author, after written approval by the Internal Scientific Committee. Requests to access these datasets should be directed to Internal Scientific Committee (info@pedianet.it).

## Supplementary Materials

The following supporting information can be downloaded at: https://www.mdpi.com/article/10.3390/children10030547/s1, Table S1: Adverse events of medical interest related ICD9-CM codes; Table S2: Exemptions codes by pathology; Table S3: Therapy at the index date and compared the distribution of medications prescribed before and after the first prescription of ozenoxacin 1% cream observed in our cohort (29 January 2019). Pedianet 2016–2021.
