# Supplementary material for: Retrospective Analysis of the Real-World Use of Topical Antimicrobials in the Paediatric Population with Impetigo in Italy: Focus on the Role of Ozenoxacin 1% Cream
Source: Children (Basel). 2023 Mar 13;10(3):547. doi: 10.3390/children10030547 (PMC10047292; doi:10.3390/children10030547)
Supplement: Supplementary file 1 [file children-10-00547-s001.zip › children-2157811-supplementary.pdf]

Supplementary material

Table S1. Adverse events of medical interest related ICD9-CM codes.

Event	ICD9CM
Itch	698.2, 698.8, 698.9
Erythema/Skin rash	695.1, 782.1,
Dermatitis/Eczema	698.4, 692.3, 692.9, 782.8
Edema	782.3, 782.2,
Urticaria/ Skin vescicular	708.0, 708.3, 708.9,
Pain at the application site	682.0, 780.99
Generic allergic reaction	995.3
Hypertrichosis	704.1, 704.2,
Hypopigmentation	709.0,
Skin atrophy	701.8

Table S2. Exemptions codes by pathology

Description	Frequency	Percent
Anoressia nervosa, bulimia	1	0.76
Asma	20	15.27
Altre Malformazioni Del Cuore	1	0.76
Anomalie Del Bulbo Cardiaco E Anomalie Del Setto Cardiaco	1	0.76
Aritmie Cardiache	1	0.76
Artrite Reumatoide Giovanile Monoarticolare	1	0.76
Artrite Reumatoide Giovanile Pauciarticolare	1	0.76
Asma Cronico Ostruttiva	1	0.76
Diabete Mellito	3	2.29
Epilessie	4	3.05
Ipotiroidismo Acquisito (Grave)	1	0.76
Ipotiroidismo Congenito (Grave)	1	0.76
Malattia Cardiopolmonare Cronica	2	1.53
Malattia Celiaca	8	6.11
Malattia della Valvola Aortica + Mitrale.	1	0.76
Malattia della Valvola Mitrale.	1	0.76
Malattie Della Valvola Aortica	2	1.53
Malattie Di Altre Strutture Endocardiche	2	1.53
Malformazione del Bulbo Cardiaco e Setti Intracard..	2	1.53
Malformazioni Cardiache (Altre Malf.).	1	0.76
Neonati Prematuri, Immaturi, A Termine Con Ricovero In Terapia Intensiva Neonatale	63	48.09
Sindrome di Down	1	0.76

Soggetti Affetti Da Patologie Neoplastiche Maligne E Da Tumori Di Comportamento Incerto	4	3.05
Soggetti Nati Con Condizioni Di Gravi Deficit Fisici, Sensoriali E Neuropsichici	8	6.11
TOTALE	131	100

As previously mentioned, treatment duration was calculated in two ways, depending on whether the duration of systemic antibiotic treatment was considered or not.

As an example, considering two patients, one who was prescribed only a topical antibiotic, and one who was prescribed both a topical and later a systemic antibiotic as shown in the table below.

		Prescription date	End of prescription date	Duration
Patient 1	Topical antibiotic	01/01	05/01	4 days
Patient 2	Topical antibiotic	01/01	07/01	6 days
	Systemic antibiotic	05/01	12/01	7 days

Treatment duration for patient 1 would be 4 days with both calculating methods.

For patient 2, treatment duration using the first method would be 6 days (duration for the topical antibiotic), and treatment duration using the second method would be 11 days ("total treatment duration", 12/01 – 1/01).

Table S3. Therapy at the index date and compared the distribution of medications prescribed before and after the first prescription of ozenoxacin 1% cream observed in our cohort (29 Jan 2019). Pedianet 2016-2021

**Prescription at index date**	**Pre-** N (%)	**Post-** N (%)
D01AC01 – clotrimazole	16 (0.8)	15 (1.2)
D01AC02 – miconazole	4 (0.2)	2 (0.2)
D01AC03 – econazole	1 (0.1)	4 (0.3)
D01AC07 – tioconazole	1 (0.1)	3 (0.2)
D01AC08 – ketoconazole	1 (0.1)	1 (0.1)
D01AC10 – bifonazole	3 (0.2)	3 (0.2)
D01AC20 - imidazoles/triazoles in combination with corticosteroids	2 (0.1)	1 (0.1)
D06AA02 - chlortetracycline	38 (2)	3 (0.2)
D06AX01 – fusidic acid	731 (37.8)	263 (20.8)
D06AX07 – gentamicin	254 (13.1)	90 (7.1)
D06AX09 – muciprocin	347 (18)	328 (26)
D06AX12 – amikacin	17 (0.9)	3 (0.2)
D06AX13 - retapamulin	34 (1.8)	5 (0.4)
D06AX14 - ozenoxacin		256 (20.3)
D06AX15 - rifamycin		9 (0.7)
D07CA01 - hydrocortisone and antibiotics	30 (1.6)	20 (1.6)
D07CB01 - triamcinolone and antibiotics	83 (4.3)	23 (1.8)
D07CC01 - betamethasone and antibiotics	333 (17.2)	216 (17.1)
D07CC04 - beclometasone and antibiotics	37 (1.9)	16 (1.3)
D07CD01 - clobetasol and antibiotics		1 (0.1)
